# Gains and losses of coral skeletal porosity changes with ocean acidification acclimation

**DOI:** 10.1038/ncomms8785

**Published:** 2015-07-17

**Authors:** Paola Fantazzini, Stefano Mengoli, Luca Pasquini, Villiam Bortolotti, Leonardo Brizi, Manuel Mariani, Matteo Di Giosia, Simona Fermani, Bruno Capaccioni, Erik Caroselli, Fiorella Prada, Francesco Zaccanti, Oren Levy, Zvy Dubinsky, Jaap A. Kaandorp, Pirom Konglerd, Jörg U. Hammel, Yannicke Dauphin, Jean-Pierre Cuif, James C. Weaver, Katharina E. Fabricius, Wolfgang Wagermaier, Peter Fratzl, Giuseppe Falini, Stefano Goffredo

**Affiliations:** 1 Department of Physics and Astronomy, University of Bologna, Viale Berti Pichat 6/2, 40127 Bologna, Italy; 2 Centro Enrico Fermi, Piazza del Viminale 1, 00184 Rome, Italy; 3 Department of Management, University of Bologna, Via Capo di Lucca 34, 40126 Bologna, Italy; 4 Department of Civil, Chemical, Environmental, and Materials Engineering, University of Bologna, Via Terracini 28, 40131 Bologna, Italy; 5 Department of Chemistry ‘G. Ciamician', University of Bologna, Via F. Selmi 2, 40126 Bologna, Italy; 6 Department of Biological, Geological and Environmental Sciences, Section of Geology, University of Bologna, Piazza di Porta S. Donato 1, 40126 Bologna, Italy; 7 Marine Science Group, Department of Biological, Geological and Environmental Sciences, Section of Biology, University of Bologna, Via F. Selmi 3, 40126 Bologna, Italy; 8 The Mina and Everard Goodman Faculty of Life Sciences, Bar-Ilan University, Ramat-Gan 52900, Israel; 9 Section Computational Science, Faculty of Science, University of Amsterdam, Science Park 904, room C3.147, 1090 GE Amsterdam, The Netherlands; 10 Institute of Materials Research, Helmholtz-Zentrum Geesthacht, Outstation at DESY, Building 25c Notkestr. 85, D-22607 Hamburg, Germany; 11 Micropaléontologie, UFR TEB Université P. & M. Curie, 75252 Paris, France; 12 Wyss Institute for Biologically Inspired Engineering at Harvard University, 60 Oxford Street, Cambridge, Massachusetts 02138, USA; 13 Australian Institute of Marine Science, PMB 3, Townsville, 4810 Queensland, Australia; 14 Department of Biomaterials, Max Planck Institute of Colloids and Interfaces, 14424 Potsdam, Germany

## Abstract

Ocean acidification is predicted to impact ecosystems reliant on calcifying organisms, potentially reducing the socioeconomic benefits these habitats provide. Here we investigate the acclimation potential of stony corals living along a pH gradient caused by a Mediterranean CO_2_ vent that serves as a natural long-term experimental setting. We show that in response to reduced skeletal mineralization at lower pH, corals increase their skeletal macroporosity (features >10 μm) in order to maintain constant linear extension rate, an important criterion for reproductive output. At the nanoscale, the coral skeleton's structural features are not altered. However, higher skeletal porosity, and reduced bulk density and stiffness may contribute to reduce population density and increase damage susceptibility under low pH conditions. Based on these observations, the almost universally employed measure of coral biomineralization, the rate of linear extension, might not be a reliable metric for assessing coral health and resilience in a warming and acidifying ocean.

## Abstract (web summary)

Global warming and ocean acidification impact coral ecosystems. Here, the authors show higher skeletal porosity and reduced bulk density at lower pH in corals living along a natural pH gradient in the Mediterranean, which may contribute to reduce population density and increase damage susceptibility.

## Graphical abstract

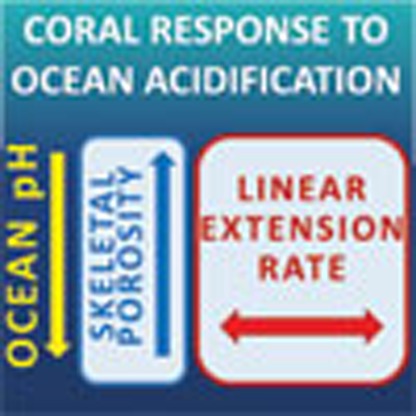

Climate change is among the biggest threats to the health of marine ecosystems. Rising atmospheric carbon dioxide partial pressure (pCO_2_)^1^ increases ocean surface pCO_2_ due to CO_2_ diffusion across the air-water interface, leading to ocean acidification (OA)^2^. Since global warming and OA are coupled and are predicted to act synergistically^3^,^4^, the future health of marine ecosystems and their corresponding long-term economic impacts on human coastal populations remain uncertain^5^,^6^,^7^. It is therefore of great interest to understand how increasing atmospheric CO_2_ concentrations will affect these marine habitats and the species that inhabit them.

Since the early 1800s, ocean pH has decreased from 8.2 by *ca.* 0.1 U^8^ and, if CO_2_ emissions continue at their current rates, the average sea surface pH is predicted to drop to 7.8 by the year 2100 (ref. 1). The Mediterranean Sea, with its closed circulation patterns and limited water exchange with the adjacent Atlantic Ocean, has already undergone a larger decrease in surface pH compared with the global average^9^, making it an ideal site for OA studies^10^,^11^,^12^,^13^.

Near Panarea Island, off the southwestern coast of Italy, lies a series of active volcanic vents in the seabed releasing CO_2_ emissions that acidify the surrounding seawater, making this location an ideal natural laboratory for OA studies. The underwater CO_2_ emissions generate a stable pH gradient with levels matching several Intergovernmental Panel on Climate Change (IPCC) sea surface pH predictions associated with different atmospheric CO_2_ emission scenarios for the end of the century^1^.

The present study investigates the effects of environmental pH on skeletal structures and growth at multiple length scales in the solitary scleractinian coral *Balanophyllia europaea* living along the pH gradient. We studied 74 corals of similar age (mean age of 12 years) that had spent their lives at the CO_2_-pH gradient. Using a combination of scanning electron microscopy (SEM), atomic force microscopy (AFM), small-angle X-ray scattering (SAXS), micro computed tomography (μCT), nanoindentation, hydrostatic weight measurement and time-domain nuclear magnetic resonance (TD-NMR), we document the skeletal mass, bulk volume, pore volume, porosity, biomineral density, bulk density, hardness, stiffness (ratio between elastic stress and strain), biometry data, net calcification rate and linear extension rate for each coral. Weight measurements combined with TD-NMR data represent a non-destructive technique for quantifying skeletal porosity over length scales spanning from tens of nanometres to tens of micrometres^14^,^15^, whereas μCT analysis permits a detailed large-scale quantitative 3D analysis of skeletal architecture, including the interseptal regions.

We show that in response to depressed calcification at lower pH, corals increase their skeletal porosity maintaining constant linear extension rate, which is important for reaching critical size at sexual maturity. However, higher skeletal porosity and reduced bulk density and stiffness may contribute to reduced mechanical strength, increasing damage susceptibility, which could result in increased mortality in an acidic environment.

## Results

### Study site and seawater carbonate chemistry

**Figure 1:**
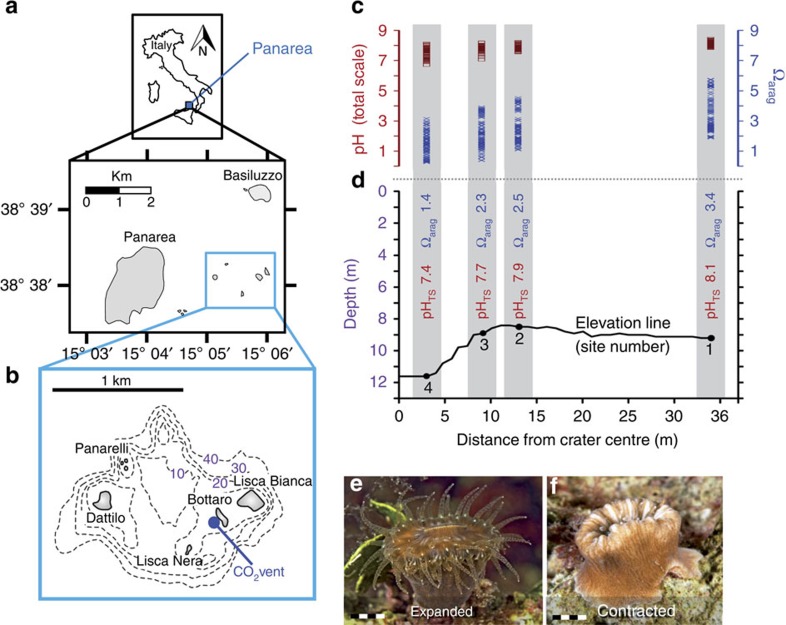
Study location. Located off the southwestern coast of Italy (**a**), near Panarea Island (**b**), there are underwater volcanic vents releasing persistent gaseous emissions (98–99% CO_2_ without instrumentally detectable toxic compounds), resulting in a stable pH gradient. Four sites at various distances from the primary vent were initially selected for study. No temperature difference exists among the four study sites throughout the year. (**c**) Ranges of measured pH_TS_ (number of observations [*n*]=103–110 per site) and Ω_arag_ (*n*=96–104 per site) values at the four sites, showing consistent increases in both pH_TS_ and Ω_arag_ from the vent crater to its periphery. (**d**) Bathymetric profile of the sites with associated mean pH_TS_ and Ω_arag_ values. No corals were found at Site 4, characterized by the lowest pH (mean pH_TS_ 7.4). Living specimens of *Balanophyllia europaea*, photographed at night with expanded tentacles (**e**) and during the day with contracted tentacles (**f**); marker 5 mm.

The four sites around the main vent are reported in Fig. 1. Site 1 (S1) has an average total scale pH (pH_TS_) of 8.1, equivalent to the average surface pH of modern oceans. S2's average pH_TS_ of 7.9 aligns with IPCC's predictions of a conservative CO_2_ emissions scenario (Representative Concentration Pathway (RCP6.0)), and the average pH_TS_ of 7.7 for S3 fits the predictions of the ‘business-as-usual' CO_2_ emissions scenario (RCP8.5). Since no corals were found at S4 (within the vent crater, pH_TS_ 7.4), only S1–3, which had growing coral populations and pH_TS_ values ranging from 8.1 to 7.7, were included in the present study. Of the measured parameters at the three sites along the pH gradient (pH_TS_, total alkalinity, temperature and salinity), only pH_TS_ differed significantly across sites (*N*=103–110; *P*<0.001, Kruskal–Wallis *χ*^2^-test). The changing pH was accompanied by significant shifts in carbonate–bicarbonate equilibria, with aragonite saturation (Ω_arag_) decreasing by >30% from S1 to S3 (Fig. 1, Supplementary Table 1, Supplementary Note 1).

### Multi-scale analysis of coral skeletal properties

**Figure 2:**
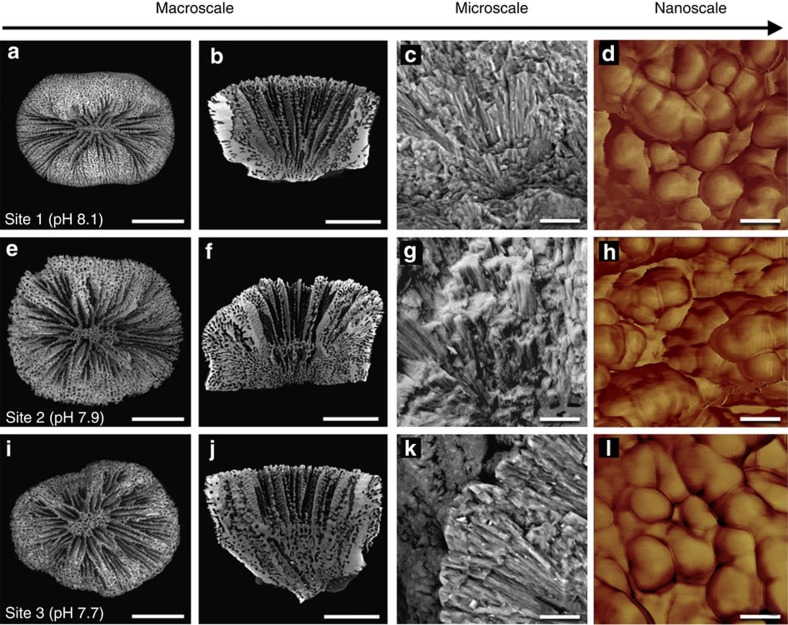
Skeletal morphology of *Balanophyllia europaea* growing under different pH conditions from the macroscale to the nanoscale. Each row in the figure corresponds to a different study site and sample age is 9–11 years. Images are representative of all observed skeletons. (**a**,**e**,**i**) Low magnification SEM images of coral skeletons, marker 5 mm. (**b**,**f**,**j**) Internal sections of corallites from μCT images, marker 5 mm. (**c**,**g**,**k**) SEM images of entire skeletal fibres from fractured septae, marker 10 μm. (**d**,**h**,**l**) AFM images of mineral grains on the skeletal fibre surfaces, marker 50 nm.

Combined results of SEM, μCT, AFM and TD-NMR skeletal analyses of corals growing at each study site revealed the detailed, multi-scale structural organization of the skeletons of *B. europaea* (Fig. 2, Supplementary Figs 1–3).

**Figure 3:**
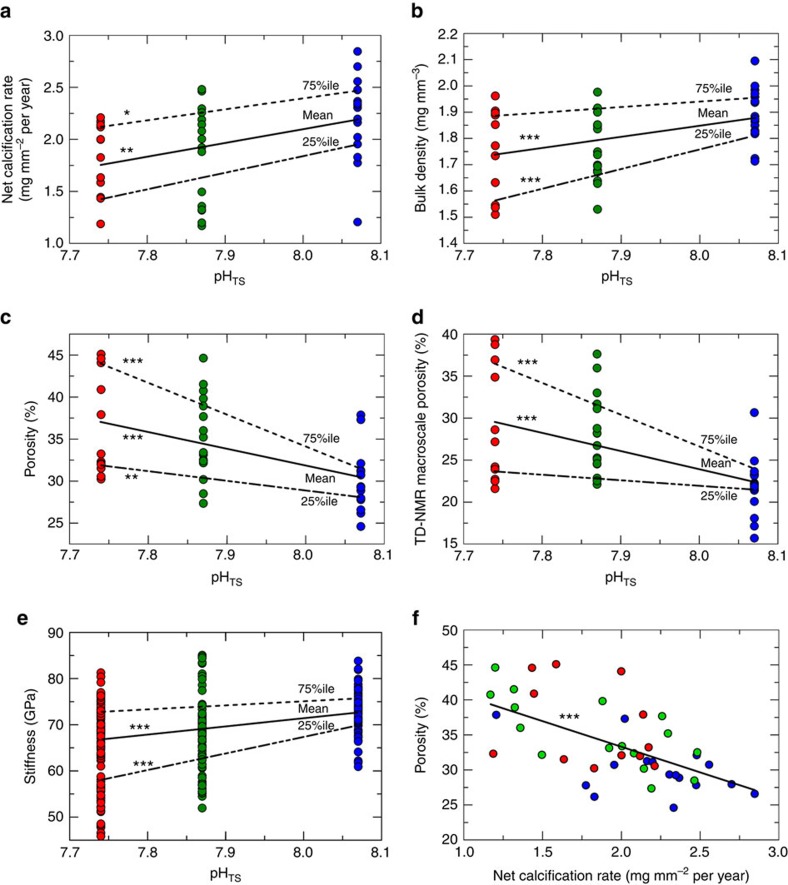
Scatterplots of skeletal parameters, and correlation analysis between porosity and net calcification rate. Site 1=blue, Site 2=green, Site 3=red. Straight lines represent the best-fit linear regression (mean, solid line), 25% quantile and 75% quantile (dashed lines). (**a**–**e**) Skeletal parameters (*y*-axes) plotted against pH_TS_. (**f**) Scatterplot of porosity (*P*_A_) versus net calcification rate in corals from Sites 1 to 3. For **a**–**d**,**f** *N*=44; for **e** *n*=122. ***P<0.001; **P<0.01; *P<0.05, robust *t*-statistics test.

At the macroscale (relating to feature sizes >10 μm), linear extension rate (averaging *ca.* 1 mm per year) did not vary among sites (Supplementary Table 2), whereas net calcification rate (*N*=44; *P*<0.01, robust *t*-statistics test) and skeletal bulk density (*N*=44, *P*<0.001, robust *t*-statistics test) significantly declined (Fig. 3, Supplementary Table 3) and skeletal porosity and macroscale porosity increased (*N*=44; *P*<0.001, robust *t*-statistics test, Fig. 3, Supplementary Fig. 1, and Supplementary Table 3). The differences between S1 and S3 were *ca.* −18% for net calcification rate, *ca.* −7% for bulk density, *ca.*+21% for porosity and *ca.*+30% for macroscale porosity (Supplementary Table 2). The corallite interseptal volume fraction from μCT (Supplementary Fig. 4) showed a difference among sites (*N*=30; *P*<0.05, F and Kruskal–Wallis *χ*^2^-tests, Supplementary Table 4) but no significant dependence on pH (Supplementary Fig. 5). The biometric data for the corallites (Supplementary Table 5) did not vary among sites.

At the micro/macroscale, skeletal stiffness significantly declined with decreasing pH (*n*=122; *P*<0.001, robust *t*-statistics test, Fig. 3, Supplementary Fig. 6, Supplementary Tables 4 and 6), whereas skeletal microscale porosity did not vary among sites (Supplementary Table 2 and Supplementary Fig. 1).

At smaller length scales (at the micro and nanoscales), SEM and AFM showed that the organization of the aragonite fibre bundles (Fig. 2c,g,k) and the morphology and packing of the constituent mineral grains (Fig. 2d,h,l) appeared similar among corals from the three different sites, confirming that the basic biomineralization products were not affected by reduced pH^13^ (Fig. 2 and Supplementary Fig. 2). Skeletal biomineral hardness did not change among sites (Supplementary Fig. 7, Supplementary Table 4). Also, skeletal biomineral density values were similar across sites and consistent with those measured in previous studies^16^ (Supplementary Table 2). Similar results were obtained from both SAXS and TD-NMR analyses, which revealed that nanoscale porosity did not change significantly with changing pH (Supplementary Figs 1e and 8–10 and Supplementary Tables 2 and 4).

The principal component analysis identified three major components: growth, skeletal porosity and biomineral density (Supplementary Tables 7 and 8). Only the first two components depended significantly on pH (Supplementary Table 9).

In summary, skeletal nano and microstructural features, linear extension rate, interseptal volume fraction and corallite biometry of *B. europaea* did not change significantly with decreasing pH, despite a clear reduction in net calcification rates. This reduction in net calcification rate was accompanied by an increase in skeletal porosity (Fig. 3f, *N*=44; *P*<0.001, robust *t*-statistics test) and a consequent decrease in skeletal bulk density and stiffness.

## Discussion

Results of the present study complement previous research on *B. europaea* at this same vent site, which revealed no changes in skeletal calcium carbonate polymorph, organic matrix content, aragonite fibre thickness and skeletal hardness in corals growing along the pH gradient^13^. There was, however, a significant reduction in population density along the pH gradient, decreasing by a factor of 3 with increasing proximity to the vent crater centre (that is, from S1 to S3)^13^.

**Figure 4:**
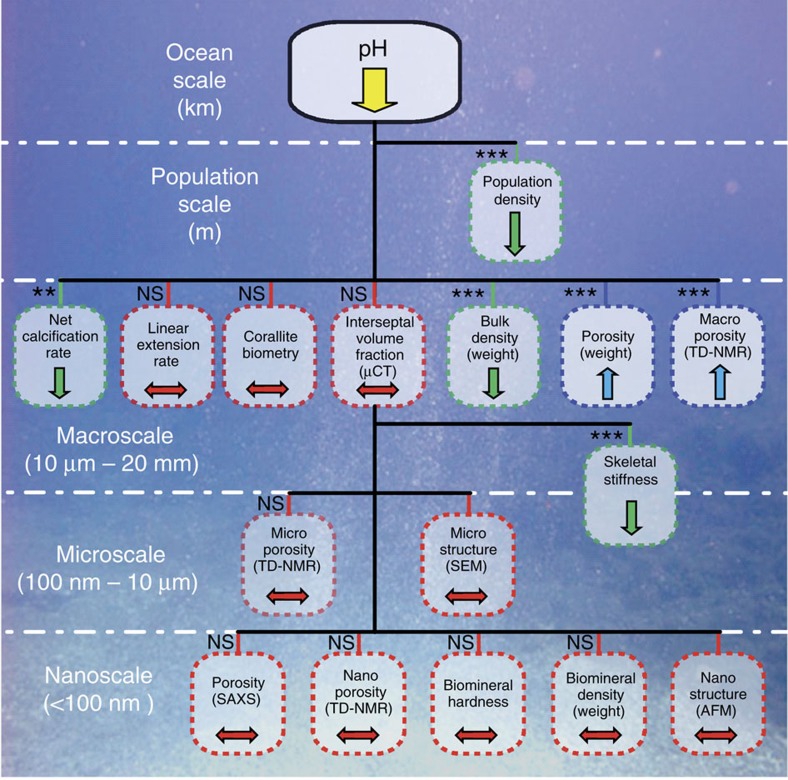
Summary of responses in *Balanophyllia europaea* skeletal parameters as a function of pH from the ocean to the nanoscale. The significant decline in population density with pH was measured in previously published research^13^. Net calcification rate and bulk density decrease with decreasing pH, whereas porosity (*P*_A_) increases, preserving the linear extension rate and corallite shape (biometry and interseptal volume fraction as measured by μCT do not correlate with pH). The increase in porosity is associated with a decrease in skeletal stiffness. At the nanoscale, the ‘building blocks' (the fundamental structural components of the coral skeleton, aragonite fibre bundles and their constituent mineral grains) produced by the biomineralization process are substantially unaffected by increased acidity. Green boxes denote parameters found to have a direct relationship with pH, blue boxes denote parameters that have an inverse relationship and red boxes denote parameters found to have no relationship with pH. The significances of the regression of each parameter (dependent variable) with pH (independent variable) are indicated; ***P<0.001; **P<0.01; NS indicates no significance. Micro and nanoscale structure observations by SEM and AFM represent qualitative data.

Figure 4 summarizes these results at the ocean, population, macro, micro and nanoscales for *B. europaea*. At the macroscale, increasing acidity was associated with a reduction in net calcification rate and a parallel increase in skeletal porosity, coupled with a decrease in skeletal bulk density. Linear extension rate and corallite shape (biometry and interseptal volume fraction) did not depend on pH, probably as a result of the compensation of reduced net calcification rate by increased skeletal porosity. At the micro/macroscale, the declining skeletal stiffness with decreasing pH could be coupled to an increased volume fraction of pores having a size comparable to the indentation area (that is, at the border between the micro and macroscales). At the nanoscale, porosity, biomineral hardness and density were not significantly affected by pH. These results, bolstered by qualitative SEM and AFM analyses, suggest that the ‘building blocks' produced by the biomineralization process are substantially unaffected, but the increase in skeletal porosity is both a gain and a loss for the coral. In fact, in an acidic environment, where the net calcification is depressed, enhanced macroporosity keeps linear extension rate constant, potentially meeting functional reproductive needs (for example, the ability to reach critical size at sexual maturity); however, it also reduces the mechanical strength of the skeletons, increasing damage susceptibility, which could result in increased mortality and the observed population density decline^13^.

While the results reported here for *B. europaea* may not be representative of the generalized response of all coral species to OA, they are consistent with field observations made on other reef-building scleractinians. For example, while maintaining constant skeletal linear extension, decreased rates of calcification and losses in bulk skeletal density as a function of reduced aragonite saturation have been observed in *Montasraea annularis*^17^ and *Porites astreoides*^18^. While low aragonite saturation as a sole driver for the observed reduction in coral calcification has been discussed^19^, our conclusions regarding a balance between reduced net calcification rate and increased macroporosity to maintain constant linear extension can explain the outcomes of those studies^17^,^18^. In fact bulk density depends both on biomineral density and porosity. Our multi-scale analysis shows that all the skeletal features at the nano and microscales, including biomineral density, are unchanged. The decrease of bulk density with decreasing pH depends on the increase of macroporosity, leaving the linear extension rate constant.

Our findings, together with the well-described detrimental effects of heat stress on the scleractinian zooxanthellate coral *B. europaea*^16^,^20^,^21^,^22^,^23^, provide several independent and consistent clues regarding the sensitivity of this species to global climate change predicted for the coming decades. Together with results from previous studies^24^, we demonstrate that the almost universally employed measure of coral biomineralization, namely the rate of linear extension, might not be a reliable metric for assessing coral health and resilience in a warming and acidifying ocean. Indeed, although the coral's ability to maintain linear extension rate and gross skeletal morphology under conditions of decreasing oceanic pH could allow it to reach sexual maturity, it could reduce skeletal resistance to environmental challenges, affecting the long-term survivability of the species.

## Methods

### Study site

Off the southwestern coast of Italy, near the island of Panarea, an area delimited by the islets of Dattilo, Bottaro, Lisca Nera and Lisca Bianca (Fig. 1) is characterized by underwater volcanic CO_2_ vents. The main vent, a crater measuring 20 m × 14 m and ∼10-m deep, generates a persistent column of CO_2_ bubbles that rise from the seabed to the sea surface. In this hydrothermally stable setting with ambient temperature, CO_2_ emissions establish a pH gradient that extends ∼34 m from the centre of this crater to its periphery^13^. Three sites along this pH gradient were chosen for study. Distances (*d*) from the main crater centre and mean pH_TS_ values of the three sites from which corals were collected are: S1 (the control site), *d*=34 m, pH_TS_=8.07; S2, *d*=13 m, pH_TS_=7.87; S3, *d*=9 m, pH_TS_=7.74. Water depth varies from 11.6 m within the crater to 8.8 m at the crater edge (S2) to 9.2 m at the outer margins (S1), where the local pH matches that of the surrounding seawater. The study site has stable hydrothermal–chemical properties^25^.

### The corals

*B. europaea* (Fig. 1) is a solitary zooxanthellate scleractinian coral endemic to the Mediterranean Sea found at depths ranging from 0 to 50 m^26^. Specimens of *B. europaea* were randomly collected by SCUBA diving at the three study sites along the pH gradient (26 from S1, 26 from S2 and 22 from S3) between November 2010 and May 2013. This sample size was chosen to limit damage on the natural population, which significantly diminishes in the most acidic sites^13^, and was considered suitable for properly describing the skeletal properties, as shown in previous studies^13^,^14^. Biometric data were recorded for the specimens (that is, width-to-length, width-to-height and height-to-length ratios, Supplementary Table 5). Nanoindentation, hydrostatic weight measurement, SEM, AFM, SAXS, μCT and TD-NMR analyses were performed on a subsample of specimens from each site.

### Coral sample cleaning

Coral skeletons were submerged in 1% solution of sodium hypochlorite for 3 days to dissolve polyp tissue. After washing with deionized water and drying at 50 °C for 3–4 days, each coral was examined under a binocular microscope to remove fragments of sediment, rock and encrusting organisms^16^.

### Weight measurements

The buoyant method, usually applied for corals^27^,^28^,^29^, was used to measure the total volume occupied by the coral skeleton (called bulk volume, *V*_B_), the bulk density (*d*_b_, ratio of the mass to *V*_B_), the biomineral density (*d*_r_, ratio of the biomineral mass to biomineral volume, excluding pore volume connected to the external surface, also called real density or micro-density) and the apparent porosity (or effective or connected porosity, *P*_A_)^30^ (ratio of the pore volume connected to the external surface (*V*_A_) to *V*_B_). This method is based on the Archimedean principle applied to a specimen after full saturation with the same fluid in which it is submerged (water in our case). It is worth to underline that the pore volume (*V*_A_) measured by the buoyant method is only the volume of the pores that can be saturated with water, that is, connected with the external surface. Pores inside the biomineral that are not connected to the external surface (occluded pores) are not measured. An estimate of the occluded porosity gave a negligible maximum value of ∼3% (in porosity units) (Supplementary Note 2), which was homogeneous among sites. For coherence with coral literature, for the apparent porosity we use the term porosity.

The skeletons were weighed with a precision balance to determine the dry mass (*m*) and then placed inside a dryer chamber and evacuated with a rotary mechanical pump down to a vacuum of 10^−2^ mbar. After 6 hours, water was gently introduced to fully saturate the samples. The pump was switched off, the chamber was vented to the ambient atmosphere, and the masses of the fully water-saturated samples (*m*_s_) determined. With a hydrostatic balance, the masses of saturated samples fully immersed in water (*m*_h_) were determined. The skeletal parameters were calculated (Supplementary Table 2) by means of the following operational definitions: *ρ*_w_=water density, *V*_A_=(*m*_s_−*m*)/*ρ*_w_; *V*_B_=(*m*_s_−*m*_h_)/*ρ*_w_; *P*_A_=*V*_A_/*V*_B_=(*m*_s_−*m*)/(*m*_s_−*m*_h_); *d*_b_=*m*/*V*_B_; *d*_r_=*m*/(*V*_B_−*V*_A_).

After weight measurement, the fully saturated samples were removed from the water container and rapidly placed on wet paper to remove excess water on the external surface. Each specimen was then placed in the bottom of a glass tube, which was then sealed for TD-NMR measurements.

### TD-NMR method and parameter definitions

This technique (Supplementary Note 3) was applied to obtain skeletal ‘pore-size' distributions by the analysis of quasi-continuous distributions of the transverse relaxation time *T*_2_ (ref. 14) by means of the algorithm UPEN^31^, implemented in the UpenWin software^32^. Supplementary Figure 1a shows examples of *T*_2_ distributions for specimens of *B. europaea*. In all cases, the slope of the distribution presented a strong increase at a specific *T*_2_ value, the ‘cutoff', revealing a sharp boundary between two distinct pore classes. This point was chosen as the boundary between smaller (shorter *T*_2_) and larger (longer *T*_2_) pores. The ratio of the area under the distribution for *T*_2_ larger than the cutoff to the total area under the distribution, called ‘macroscale pore volume fraction' (Supplementary Fig. 1b), indicates the fraction as a percentage of the pore volume in the macroscale. On the basis of the comparison with mercury intrusion porosimetry data, the sizes of pores in the macroscale pore volume fraction, corresponding to the major fraction of pores, are >10–20 μm. The tail in the distribution is about 3 orders of magnitude long and should correspond to smaller pore sizes up to tens of nanometres^14^. The distribution with *T*_2_ <3 ms was chosen to identify the signal corresponding to water in the smallest pores or size-scale, which we refer to here as ‘nanoscale pore volume fraction'. The remaining fraction of the distribution is called ‘microscale pore volume fraction'. The nano, micro and macroscale pore volume fractions multiplied by the skeletal porosity (Supplementary Table 2) produced the TD-NMR nano, micro and macroscale porosities, respectively.

### TD-NMR apparatus and data acquisition

A homebuilt relaxometre based on a 0.2 T permanent magnet operating at 8 MHz was used for acquisition of the transverse relaxation curve of the ^1^H nuclei of water molecules saturating the cleaned coral skeletons. The relaxometer was equipped with a coil ∼2 cm in diameter to analyse the entire coral and a Spinmaster console (Stelar, Mede, Pavia, Italy) for automatic pulse sequence transmission and data acquisition. The transverse relaxation data were acquired by using the Carr–Purcell–Meiboom–Gill sequence^33^ with a 200 μs echo time. Sixteen corals from S1, 16 corals from S2 and 12 corals from S3 were analysed.

### SEM images

To investigate the meso and macroscale organization of the aragonite fibre bundles, SEM analyses were performed. The cleaned coral skeletons were mounted (uncoated) to conductive carbon tape and examined with a Tescan Vega3 GMU (Czech Republic) variable pressure scanning electron microscope. Three corals per site were analysed.

### Evaluation of net calcification rate

The age of each coral was determined based on growth band analysis via computerized tomography^22^,^34^. The linear extension rate was obtained by dividing the length of each sample (maximum axis of the oral disc) by its age. The net calcification rate was calculated as: net calcification rate=bulk density × linear extension rate^21^,^35^ (Supplementary Table 2).

### μCT analysis

To compute the corallite interseptal volume fraction (ratio of the total pore space volume inside the corallite to the total volume occupied by the corallite itself), μCT analyses were acquired and the convex hull approach was followed. μCT scans of coral skeletons were performed with a GE phoenix X-ray Nanotom S. The voxel sizes in the scans varied from 2.91 to 13.25 μm depending on the actual size of the investigated coral sample. All image processing steps in the analysis were performed with the open source software FIJI^36^. Supplementary Fig. 3 shows different views of the 3D volume renderings obtained from three corals of the same age from each of the three study sites. Ten corals from each site were analysed.

### Nanoindentation

To analyse mechanical properties of the coral skeletons, skeletal stiffness (Young's modulus) and hardness were determined by Oliver–Pharr analysis of load-depth curves^37^, using a Nanoindentation Tester, model NHT-TTX by CSM Instruments, Switzerland, equipped with a Berkovich diamond tip (opening angle=142.3°). On each sample, a minimum of 10 indentation tests were carried out, in the oral region, with a minimum distance of 30 μm between two tests (the total number of tests for each site is reported in Supplementary Table 4). To avoid influence from the embedding resin, a minimum distance of 100 μm from the skeleton/resin boundaries was always maintained.

### SAXS analysis

To obtain information on skeletal porosity at the nanoscale, SAXS analyses (Supplementary Note 4) were performed on three corals per site. Sections were mounted onto a laboratory SAXS apparatus with a rotating anode X-ray generator (Bruker, AXS, Karlsruhe, Germany) operating with a copper anode. Light microscopy images of the three specimens are shown in Supplementary Fig. 8 together with X-ray radiographies taken with the SAXS instrument.

### AFM analysis

To study the morphology and packing of skeletal mineral grains at the nanoscale, AFM images were acquired. Samples were lightly polished using diamond paste, cleaned with milli-Q water and observed using a Veeco AFM Dimension 3100 Nanoscope III, Plainview, NY. The probe consisted of a cantilever with an integrated silicon nitride tip. Samples were imaged at room temperature and in air using tapping mode phase contrast imaging.

### Statistical analysis

Statistical analyses (analysis of variance, multivariate, principal components and quantile analysis) were performed using the Statistical Package STATA 9.0 (StataCorp LP). To test the significance of the differences among sites, parametric (F) or non-parametric (Kruskal–Wallis *χ*^2^) tests were run. Non-parametric tests were performed for data that did not assume normal distributions. Multivariate regression analyses were performed to investigate the relationships between one dependent and one or several independent variables, using ordinary least squares robust to outliers. The model is described by the equation (1):


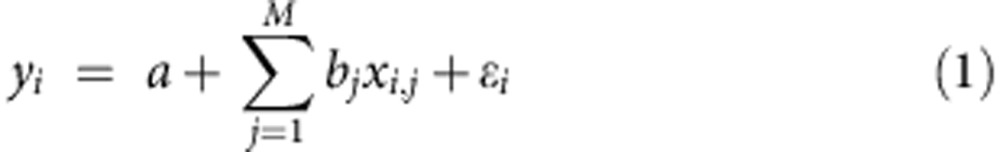


where the index *i* refers to the *i*-observation, *x*_*j*_ is an independent variable, *y*_*i*_ is the value of the dependent variable and *ɛ*_*i*_ is the corresponding error. The constants *a*, *b*_*j*_ (*j*=1, *M*) are the best-fit parameters, to be determined by the best fit. Quantile analysis was performed to study the previous relationships for homogeneous groups of values of the dependent variable. This analysis was used to give a more comprehensive picture of the effect of the independent variable (pH) on the dependent variables of Fig. 3 and Supplementary Fig. 1, as it can show different effects of the independent variable in different ranges of the dependent variable.

## Additional information

**How to cite this article:** Fantazzini, P. *et al*. Gains and losses of coral skeletal porosity changes with ocean acidification acclimation. *Nat. Commun.* 6:7785 doi: 10.1038/ncomms8785 (2015).

## Supplementary Material

Supplementary Information Supplementary Figures 1-10, Supplementary Tables 1-9, Supplementary Notes 1-4 and Supplementary References.

Supplementary Movie 1 Balanophyllia europaea close to the crater
