# Supplementary material for: Gains and losses of coral skeletal porosity changes with ocean acidification acclimation
Source: Nat Commun. 2015 Jul 17;6:7785. doi: 10.1038/ncomms8785 (PMC4518299; doi:10.1038/ncomms8785)
Supplement: Supplementary Information — Supplementary Figures 1-10, Supplementary Tables 1-9, Supplementary Notes 1-4 and Supplementary References. [file ncomms8785-s1.pdf]

Supplementary Information

SUPPLEMENTARY FIGURES

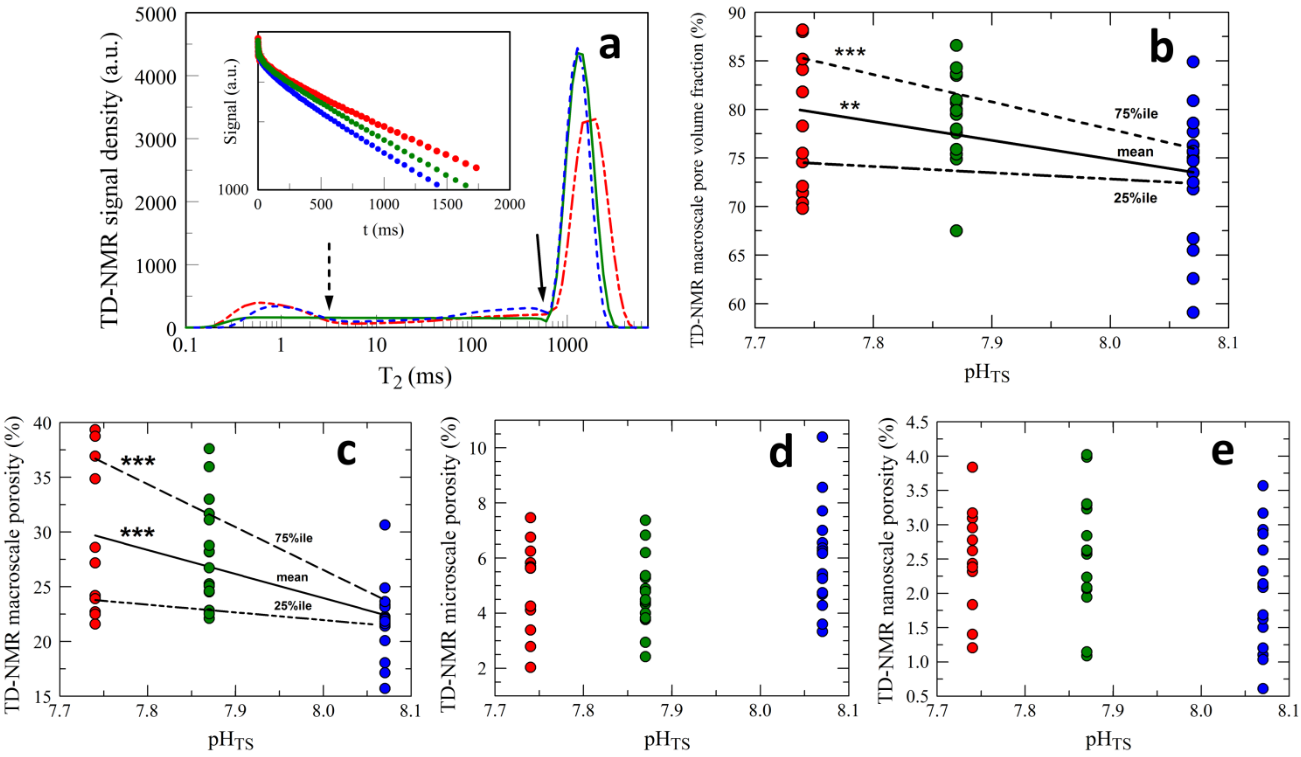

Supplementary Figure 1. ***T*$_2$ distributions and skeletal porosity analyses at different scales.** Site 1 = blue, Site 2 = green, Site 3 = red. **(a)** Examples of T_2 relaxation time distributions of ^{1}H NMR signal from cleaned skeletons of *B. europaea* after water saturation of the connected pore-space. The inset shows the corresponding experimental relaxation curves. By assuming that the smallest pores have T_2 values less than 3 ms (dashed arrow) and the largest pores have T_2 values that exceed the cut-off (solid arrow), three regions can be identified from the T_2 distributions; for simplicity, here they are referred to as nanoscale, microscale, and macroscale pore volumes. The ratios of these pore volumes to the total area under the distribution curve represent the corresponding pore volume fractions. **(b)** Macroscale pore volume fraction plotted against pH_{TS}. By knowing the porosity (P_A) of the samples, it was possible to compute the corresponding contributions to the porosity. Macro-, micro-, and nanoscale porosity, are plotted against pH_{TS} in **(c)**, **d)**, and **(e)**, respectively. The sum of macro- micro- and nanoporosity corresponds to the porosity. The dependence on pH_{TS} was stronger for corals with larger macroscale pore volume fractions, as shown by quantile analyses (N = 44; ***p<0.001 and **p<0.01, robust t-statistics test). The regressions shown in (**b**) and **(c)** are statistically significant; the regressions shown in **(d)** and **(e)** are not statistically significant.

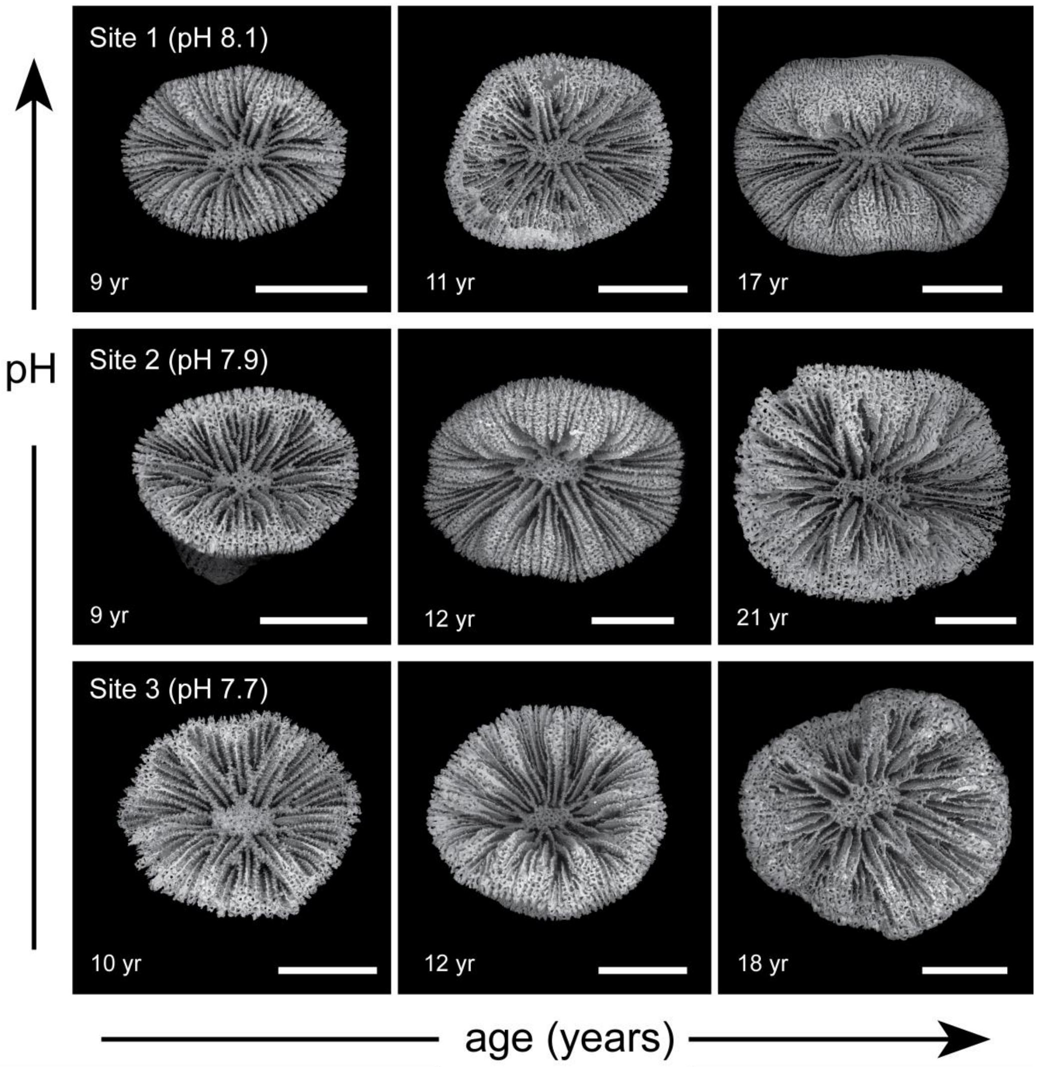

Supplementary Figure 2. **Low magnification SEM images of skeletons of corals growing in Sites 1–3.** The age of each coral, which was determined using computerized tomography to count the number of annual growth bands[1], is indicated in the bottom left corner of each image. Scale bars 5 mm.

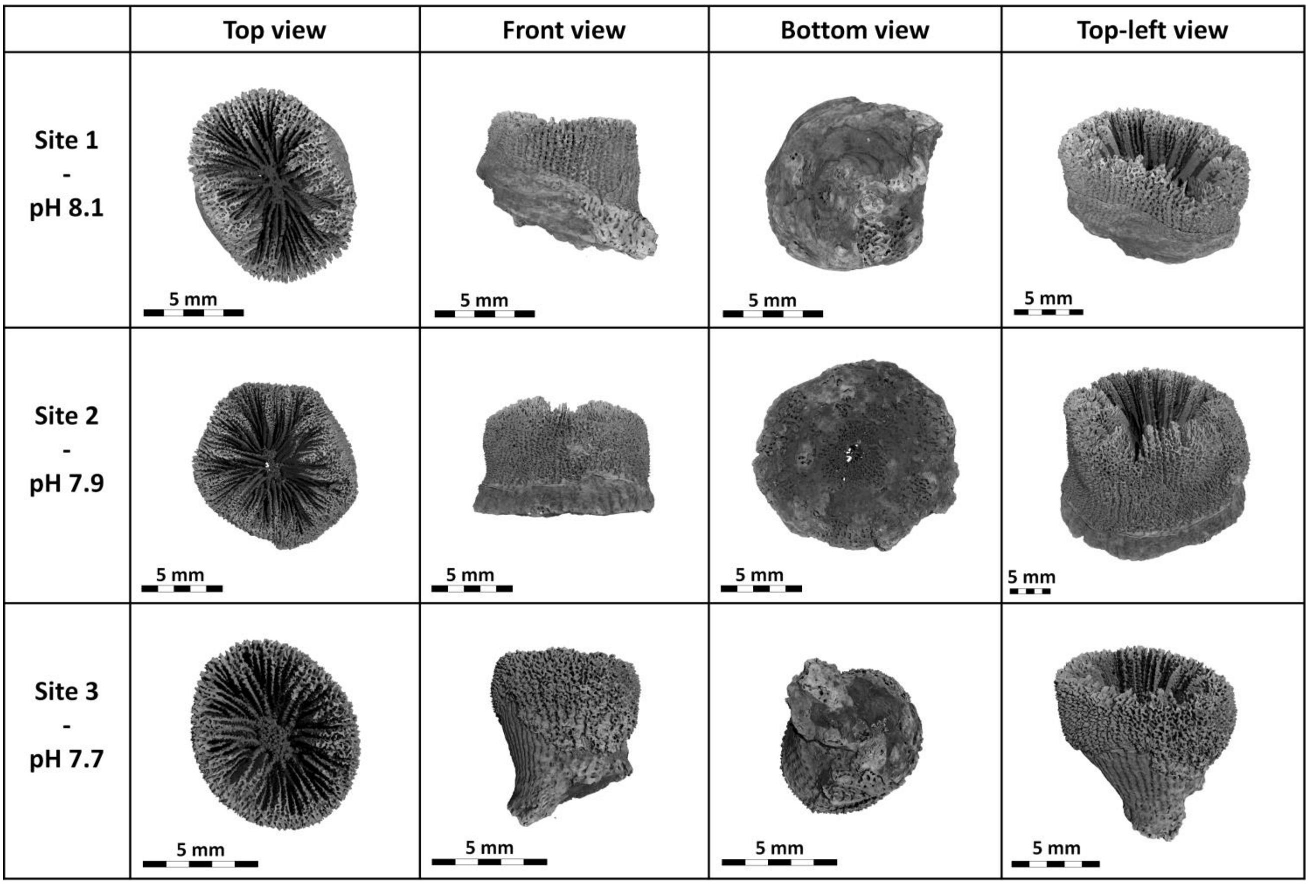

Supplementary Figure 3. **µCT images of coral skeletons.** Different views of 3D volume renderings of 10-year-old *B. europaea* samples from each study site.

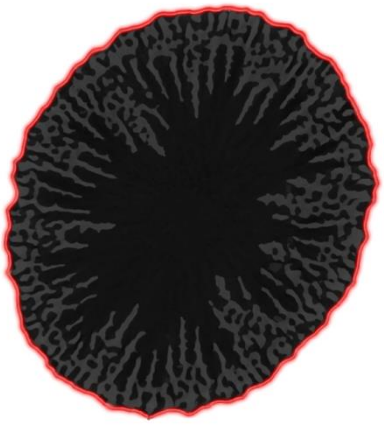

Supplementary Figure 4. **A µCT image from an image stack with fitted surface indicated by the red line for total volume calculation.** For the computed corallite interseptal volume fraction (ratio of the total pore space volume inside the corallite to the total volume occupied by the corallite itself), the volume of the coelenteron is included in the computation of the total corallite volume. A threshold was applied to the image data in order to segment the voxels belonging to the skeleton and to determine the volume of the skeleton itself.

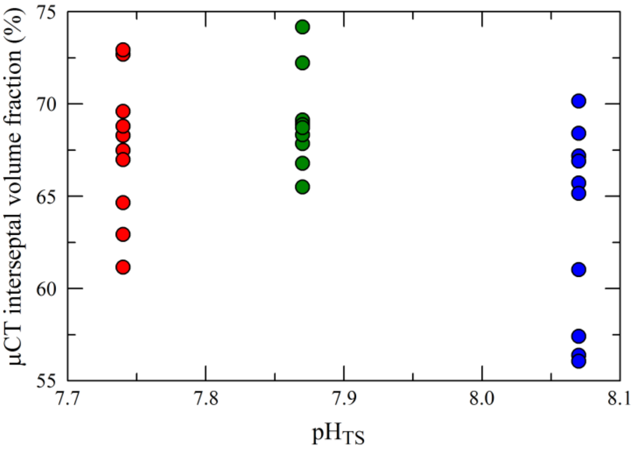

Supplementary Figure 5. **Scatterplot of corallite interseptal volume fraction determined by μCT vs pH.** Site 1 = blue, Site 2 = green, Site 3 = red.

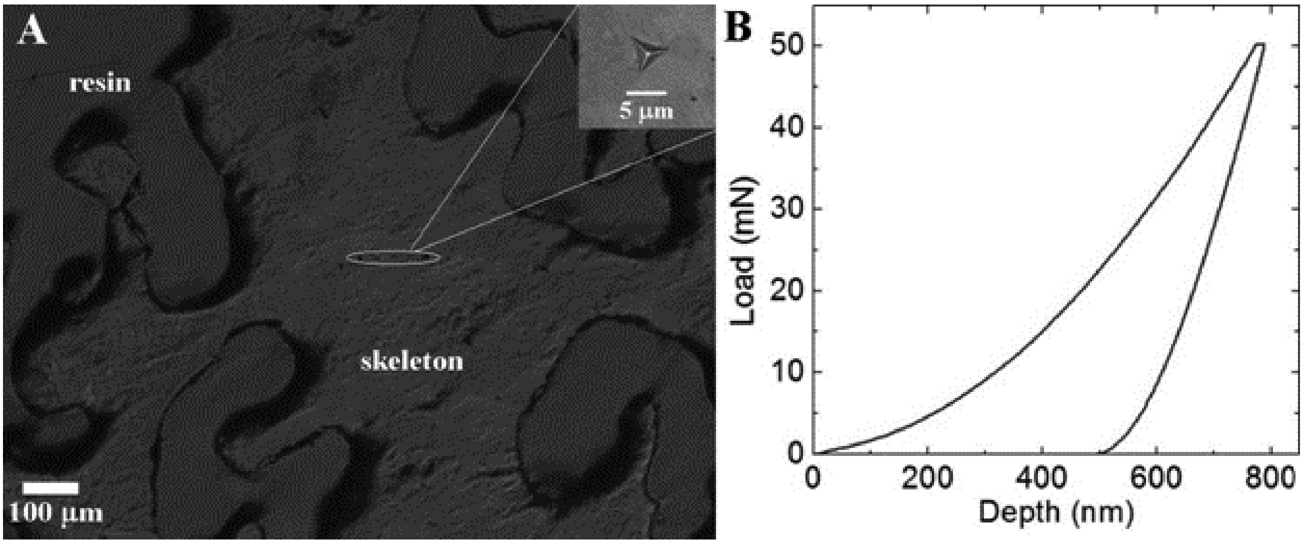

Supplementary Figure 6. **(A) Optical micrograph of a typical skeletal section used for nanoindentation measurements.** The white ellipse highlights an area with a sequence of five indentations. The top right inset displays a higher magnification view of one indentation's residual imprint. The apparent roughness arises from optical contrast between differently oriented fibers. **(B)** Typical load-depth curve at 50 mN maximum load (corresponding to a depth of about 800 nm), loading/unloading rate = 100 mN/min, hold period at maximum load = 10 s).

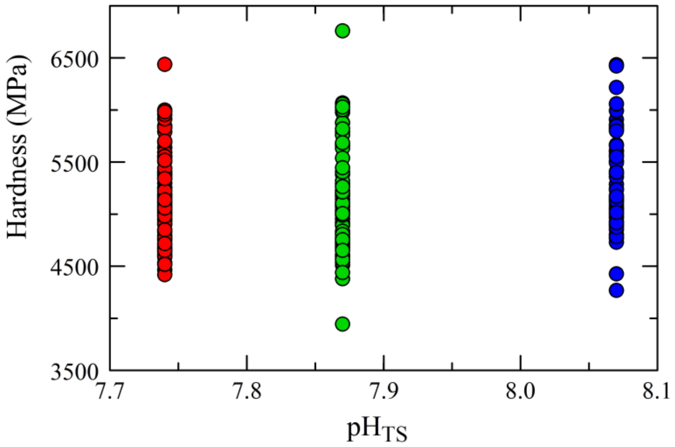

Supplementary Figure 7. **Scatterplot of skeletal hardness measured by nanoindentation in corals from the three study sites.** Site 1 = blue, Site 2 = green, Site 3 = red.

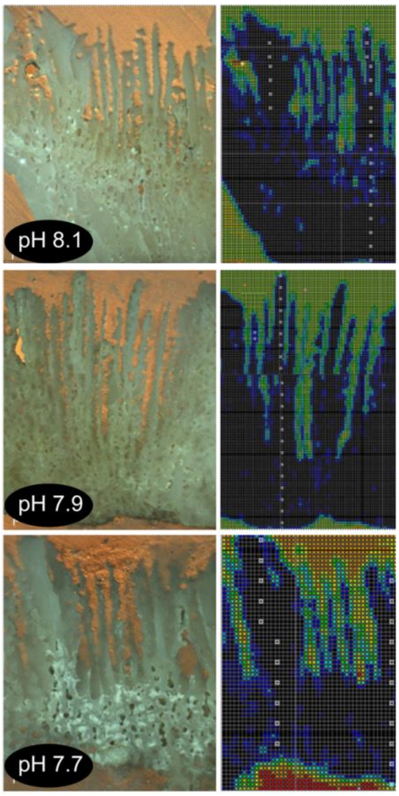

Supplementary Figure 8. **Light microscopy images on the left and corresponding X-ray radiographies on the right of corals from each of the three study sites.** White squares in the radiographies indicate positions where SAXS data were collected.

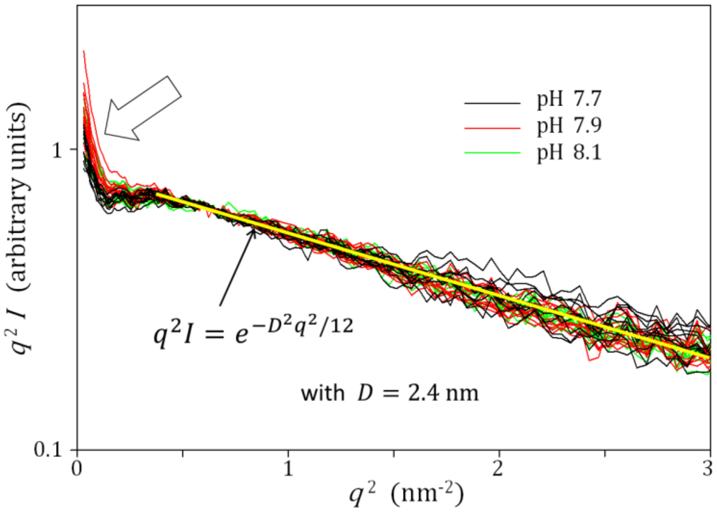

Supplementary Figure 9. **Summary of SAXS data from coral sections for the three study sites**. Most of the signal (at q larger than 1 nm^{-1}) was fully consistent at all sites and compatible with the scattering due to plate-shaped inclusions with a thickness of 2.4 nm (yellow line). After subtraction of the signal represented by the yellow line, the remaining intensity (which varied substantially across specimens and is indicated by the arrow) is attributed to nanoporosity (pores smaller than ~100 nm). The area under this curve is proportional to the total volume of these pores.

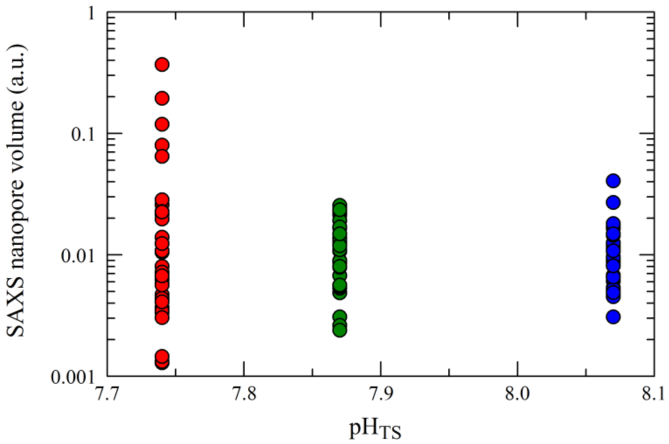

Supplementary Figure 10. **Nanopore volume obtained from SAXS measurements**. The data are in arbitrary units**.** The value on the y-axis is the integral intensity proportional to the volume of sub-100 nm pores per volume of coral skeletal material.

SUPPLEMENTARY TABLES

	pH_{TS} (total scale)	T (°C)	TA (µmol kg^{-1})	pCO_2 (µatm)	$^{(a)}HCO_3^-$ (µmol kg^{-1})	$^{(a)}CO_3^{2-}$ (µmol kg^{-1})	$^{(a)}$DIC (µmol kg^{-1})	$^{(a)}\Omega_{arag}$
**Site 1** ***d*=34 m**	8.07 (8.04–8.10)	19.2 (19.0–19.3)	2482 (2435–2529)	423 (387–460)	1923 (1888–1958)	229 (215–243)	2165 (2144–2187)	3.4 (3.2–3.7)
**Site 2** ***d*=13 m**	7.87 (7.83–7.90)	19.5 (19.3–19.7)	2478 (2424–2533)	698 (638–759)	2109 (2079–2140)	169 (157–182)	2301 (2281–2321)	2.5 (2.4–2.7)
**Site 3** ***d*=9 m**	7.74 (7.68–7.81)	19.5 (19.3–19.7)	2465 (2416–2513)	922 (764–1080)	2107 (2078–2136)	146 (135–158)	2283 (2262–2304)	2.3 (2.1–2.4)
**Statistical significance**	***	NS	NS	***	***	***	***	***

Supplementary Table 1. **Seawater carbonate chemistry data for each study site.** Data[2] are presented as mean values with 95% CIs in brackets. Site 1 is the control site, and Sites 2 and 3 are the elevated pCO_2 sites. *d* = distance in meters from the vent to the study site; T = temperature (number of observations [*n*] = 2580 per site); TA = total alkalinity (*n* = 8 per site); pCO_2 = carbon dioxide partial pressure; HCO_3^- = bicarbonate; CO_3^{2-} = carbonate; DIC = dissolved inorganic carbon; Ω_{arag} = aragonite saturation; NS = not significant; ***p<0.001, Kruskal-Wallis equality-of-populations rank test. Statistically significant results mean that values for parameters measured at both Sites 2 and 3 were significantly different from those of Site 1, and values at Site 3 were significantly different from those at Site 2 except where otherwise noted. Mean pH_{TS} values (*n* = 103–110 per site) were calculated after conversion of data to hydrogen ion concentrations. pH_{TS}, T, TA, and salinity (37‰, *n* = 107–110 per site) were used to calculate the carbonate system parameters (denoted by $^{(a)}$, *n* = 96–107 for each site).

	**Site 1: *d*=34 m** **pCO_2=423 µatm** **pH=8.07** ***N*=16**		**Site 2: *d*=13 m** **pCO_2=698 µatm** **pH=7.87** ***N*=16**		**Site 3: *d*=9 m** **pCO_2=922 µatm** **pH=7.74** ***N*=12**		
	**mean**	**se**	**mean**	**se**	**mean**	**se**	**statistical significance**
**Age (yr)**	11.1	1.3	14.5	1.6	14.8	1.9	NS
**Net calcification rate (mg $mm^{-2}yr^{-1}$)**	2.2	0.1	1.9	0.1	1.8	0.1	*
**Linear extension rate (mm yr^{-1})**	1.17	0.05	1.04	0.05	1.03	0.05	NS
**Dry mass (*m*) (mg)**	1023	237	1227	209	1229	250	NS
**Skeletal bulk volume (V_B) (mm^3)**	546	132	708	124	689	133	NS
**Skeletal bulk density (d_b) (mg mm^{-3})**	1.89	0.03	1.77	0.03	1.76	0.05	*
**Skeletal biomineral density (d_r) (mg mm^{-3})**	2.70	0.02	2.73	0.02	2.76	0.02	NS
**Skeletal porosity (P_A) (%)$^{\$}$**	30.0	0.9	35.3	1.2	36.2	1.7	**
**TD-NMR skeletal macroscale porosity (%)**	22.0	0.9	28.0	1.2	28.7	2.0	***
**TD-NMR skeletal microscale porosity (%)**	6.0	0.5	4.7	0.3	5.0	0.5	NS
**TD-NMR skeletal nanoscale porosity (%)**	2.0	0.2	2.6	0.2	2.5	0.2	NS

Supplementary Table 2. **Summary of acquired and computed skeletal parameters for *B. europaea.*** Data are presented as means and standard errors (se). *N* = number of corals; *d* = distance in meters from the vent to the study site. The values of F-test and Kruskal-Wallis χ^2 equality-of-populations rank tests suggest significant differences among sites only for net calcification rate, total skeletal porosity, bulk density, and macroscale porosity. Macro-, micro-, and nanoscale porosity values were obtained by combining total skeletal connected porosity by weight with TD-NMR data. NS = not significant; ***p<0.001, **p<0.01, *p<0.05.

	**Net calcification rate**			**Skeletal bulk density (d_b)**			**Skeletal porosity (P_A)**			**TD-NMR macroscale porosity**		
	**ols**	**q25**	**q75**	**ols**	**q25**	**q75**	**ols**	**q25**	**q75**	**ols**	**q25**	**q75**
**pH**	1.33**	1.58	1.08*	0.43***	0.79***	0.21	-20.04***	-12.57**	-39.16***	-21.98***	-7.61	-41.42***
	(2.87)	(1.77)	(2.69)	(2.94)	(4.72)	(1.12)	(-3.69)	(-2.17)	(-5.96)	(-3.77)	(-1.88)	(-7.16)
**Constant**	-8.58*	-10.80	-6.24	-1.61	-4.53***	0.28	192.1***	129.3***	347.2***	199.8***	82.78**	357.6***
	(-2.33)	(-1.53)	(-1.97)	(-1.39)	(-3.43)	(0.19)	(4.48)	(2.82)	(6.70)	(4.29)	(2.58)	(7.83)
R^2	0.164			0.171			0.245			0.262		

Supplementary Table 3. **Regression and quantile analysis**. Analysis for net calcification rate, bulk density, total skeletal porosity, and TD-NMR macroscale porosity were based on data for 44 corals (16 from Site 1, 16 from Site 2, and 12 from Site 3). The coefficients in the columns are the best-fit parameters *a* (Constant) and b_j in equation (1) (noted in the 'Statistical analysis' section) obtained by ordinary least squares (ols) and by quantile analysis at 25% (q25) and 75% (q75), respectively. R^2 is the coefficient of determination. Robust t-statistics in parentheses. ***p<0.001, **p<0.01, *p<0.05.

	**Site 1: *d*=34 m pCO$_2$=423 µatm pH=8.07**				**Site 2: *d*=13 m pCO$_2$=698 µatm pH=7.87**				**Site 3: *d*=9 m pCO$_2$=922 µatm pH=7.74**				
	N	***n***	**mean**	**se**	***N***	***n***	**mean**	**se**	***N***	***n***	**mean**	**se**	**statistical significance**
**Skeletal hardness (MPa)**	3	37	5109	51	3	37	5170	88	3	48	5171	60	NS
**Skeletal stiffness (GPa)**	3	37	70.8	0.8	3	37	66.4	1.0	3	48	63.8	1.6	***
**SAXS nanopore volume (a.u.)**	3	31	$11\ 10^{-3}$	$1\ 10^{-3}$	3	34	$12\ 10^{-3}$	$1\ 10^{-3}$	3	31	$3\ 10^{-2}$	$1\ 10^{-2}$	NS
**µCT corallite interseptal volume fraction (%)**	10	Entire corallite volume	63.4	1.7	10	Entire corallite volume	69.1	0.8	10	Entire corallite volume	66.7	1.5	*

Supplementary Table 4. **Skeletal parameters by different techniques for corals from the three study sites.** *N* = number of corals; *n* = number of observations or tests; se = standard error; *d* = distance in meters from the vent to the study site; a.u.=arbitrary units. The values of F-test and Kruskal-Wallis χ^2 equality-of-populations rank tests suggest highly significant differences among sites only for stiffness. NS = not significant; ***p<0.001, *p<0.05.

	Site 1: d=34 m pCO$_2$=423 µatm			Site 2: d=13 m pCO$_2$=698 µatm			Site 3: d=9 m pCO$_2$=922 µatm			
	N	**mean**	**se**	***N***	**mean**	**se**	***N***	**mean**	**se**	**statistical significance**
**Width-to-length ratio**	26	0.82	0.01	26	0.82	0.01	26	0.85	0.02	NS
**Height-to-length ratio**	26	0.80	0.03	26	0.74	0.04	26	0.77	0.0.2	NS
**Height-to-width ratio**	22	0.99	0.05	22	0.91	0.06	22	0.92	0.04	NS

Supplementary Table 5. **Biometric data for corals from each of the three study sites.** *N* = number of corals; se = standard error; *d* = distance in meters from the vent to the study site; NS = not significant.

	Stiffness		
	**ols**	**q25**	**q75**
**pH**	21.22***<br>(4.02)	39.09***<br>(3.60)	8.98<br>(1.36)
**Constant**	-100.47*<br>(-2.39)	-247.82**<br>(-2.90)	1.48<br>(0.03)
n	122	122	122
$\boldsymbol{R^2}$	0.116	0.133	0.01

Supplementary Table 6. **Regression and quantile analyses of skeletal stiffness versus pH.** The coefficients in the columns are the best-fit parameters *a* (Constant) and b_j to equation (1) obtained by ordinary least squares (ols) and by quantile analysis at 25% (q25) and 75% (q75), respectively. *n* is the number of observations of stiffness. R^2 is the coefficient of determination. Robust t-statistics in parentheses. *** p<0.001, **p<0.01, *p<0.05

**Component**	**Eigenvalue**	**Proportion of Variance**	**Cumulative Variance**
PC1	6.6925	0.6084	0.6084
PC2	2.1144	0.1922	0.8006
PC3	1.1515	0.1047	0.9053
PC4	0.5385	0.0490	0.9543
PC5	0.2932	0.0267	0.9809
PC6	0.1627	0.0148	0.9957
PC7	0.0265	0.0024	0.9981
PC8	0.0171	0.0016	0.9997
PC9	0.0029	0.0003	1
PC10	0.0003	0	1
PC11	$2.67\ 10^{-05}$	0	1

Supplementary Table 7. **Principal component analysis.** Eigenvalues of the principal components (PCs), proportion of variance explained by each component, and cumulative variance explained. The first three components explain 90% of the variance and are considered the three major components found among all measured parameters for the 44 corals of *B. europaea* examined by TD-NMR. PC1, PC2, and PC3 explain 61%, 19%, and 10% of the variance, respectively.

	PC1	PC2	PC3
**dry mass**	**0.3457**	-0.2574	-0.0951
**age**	**0.3699**	-0.1077	-0.1294
**Skeletal pore volume**	**0.3722**	-0.0811	-0.0438
**Skeletal bulk volume (V_B)**	**0.3603**	-0.1965	-0.0877
**Skeletal porosity (P_A)**	0.2122	**0.4870**	0.2930
**Skeletal biomineral density (d_r)**	0.1280	-0.0026	**0.7901**
**TD-NMR macroscale porosity**	0.2277	**0.5444**	0.1097
**linear extension rate**	**-0.3704**	0.0989	0.1154
**net calcification rate**	**-0.3699**	-0.0900	0.1040

Supplementary Table 8. **Factor loadings of the first three principal components.** Bold type indicates the factor loadings of the variables with a substantial loading on the PC in the column (factor loading set significant if larger than 0.35). Mass, age, skeletal bulk volume, net calcification rate, and linear extension rate have a substantial loading on PC1 (growth parameter); skeletal porosity (P_A) and TD-NMR macroscale porosity on PC2; and skeletal biomineral density on PC3.

	(1)<br>**PC1**	(2)<br>**PC2**	(3)<br>**PC3**
**pH**	-6.74*<br>(-2.43)	-3.65*<br>(-2.33)	-2.03<br>(-1.70)
**Constant**	53.27*<br>(2.43)	28.83*<br>(2.33)	16.01<br>(1.70)
R^2	0.123	0.114	0.065

Supplementary Table 9. **Regression analysis between principal components and pH**. The analysis was based on data for N = 44 corals. pH is the independent variable and principal components (PCs) are the dependent variables. The coefficients in the columns are the best-fit parameters a (constant) and b_j to equation (1). R^2 is the coefficient of determination. PC1 and PC2 depended significantly on pH (*p<0.05, robust t-statistics in parentheses), but PC3 did not.

SUPPLEMENTARY NOTES

Supplementary Note 1: Seawater carbonate chemistry

Temperature (T), salinity, total alkalinity (TA), and pH (NBS) were measured periodically from July 2010 to May 2013 with a multi-parametric probe (600R, YSI Incorporated, USA) powered from a small boat and operated by SCUBA divers. Measured pH values were converted to the total scale (pH_{TS}) using CO2SYS software (Carbon Dioxide Information Analysis Center, Oak Ridge National Laboratory, U.S. Dept. of Energy). pH (NBS scale, number of measurements [*n*] = 103–110 per site) and salinity (37‰, *n* = 107–110 per site) were measured in July 2010, September 2010, November 2010, March 2011, June 2011, July–August 2011, November–December 2011, April–May 2012, June 2012, and May 2013. Temperature values (*n* = 2580 per site) were recorded by sensors (Thermochron iButton, DS1921G, Maxim Integrated Products, USA) placed at each site. Bottom water samples for TA determination were collected at each site using sterile 120 ml syringes (two replicates per site per measurement; *n* = 8 per site) in September 2010, November 2010, March 2011, June 2011, July–August 2011, November–December 2011, April–May 2012, and June 2012. After each dive, the syringe contents were immediately transferred to labelled 100 ml amber glass bottles and fixed with saturated mercuric chloride to avoid biological alteration, and stored in darkness at 4 °C prior to analysis. TA was measured by Gran titration using a 702 SM Titrino (Metrohm AG, Italy). Certified reference materials for oceanic CO_2 measurements (Batch 121 from the Andrew Dickson Laboratory, Scripps Institution of Oceanography, UC San Diego) were used to ascertain the quality of the results obtained. Mean pH_{TS} (back-transformed hydrogen ion concentration) was calculated for each site; pH, T, salinity, and TA were used to calculate the following carbonate system parameters using CO2SYS software with standard dissociation constants[3-5]: dissolved inorganic carbon (DIC), carbon dioxide partial pressure (pCO_2), carbonate (CO_3^{2-}) and bicarbonate (HCO_3^-) content, and aragonite saturation (Ω_{arag}) (Supplementary Table 1).

Supplementary Note 2: Estimate of the occluded porosity

The estimate of the occluded porosity is necessarily based on assumptions related to the features of the biomaterial, composed by aragonite and organic matrix. For aragonite we assumed the density of the pure geogenic mineral (2.94 g/cm^3) and for the organic matrix the density of a proteic crystal (1.3 g/cm^3)[6]. In our corals the organic matrix corresponds to 2.2% wt (%) of the total mass[7]. Within these assumptions, an upper limit for the occluded porosity turned out to be about 3% (in porosity units).

This value represents an overestimation because in corals aragonite can also be present in amorphous form with a density lower than the crystalline form.

This estimate was consistent with the results of a comparison between TD-NMR relaxometry and mercury injection porosimetry data[8]. The NMR signal in *B. europaea* after desiccation is about 10% of the signal of the fully water-saturated sample. This signal can be due to water absorbed by the surface or stored in interstices from which it cannot be removed by air drying, or to water in occluded pores. If that signal is all assigned to water in occluded pores, it corresponds to an occluded porosity value of about 3-4% (in porosity units), consistent with the above estimated value. Mercury injection porosimetry, used to calibrate pore sizes, showed that the occluded pores in the samples should have sizes less than 1 μm.

Supplementary Note 3: Time-Domain Nuclear Magnetic Resonance (TD-NMR)

TD-NMR, and in particular magnetic resonance relaxometry of water ^{1}H nuclei for analyzing internally connected skeletal porosity, has several advantages compared to other methods because it is a non-destructive and non-invasive procedure, allowing intact specimens to be further analyzed with other techniques. In this work, the transverse magnetization component, with transverse relaxation time T_2, was studied. In porous media saturated by water, under the assumptions that the relaxation rate of the unconfined water is negligible and the molecular diffusion is fast enough to maintain the magnetization uniform within the pores, the distribution of T_2 corresponds to specific "pore-size" distributions. The area below each distribution is proportional to the total NMR signal and therefore is proportional to the volume of water saturating the skeletal pore volume. Supplementary Figure 1a shows examples of T_2 distributions for specimens of *B. europaea* obtained through mathematical inversion of the experimental transverse relaxation curves. In the T_2 distributions, the ordinate-labeled signal density is an approximation to $dS/(d\ \ln T_2)$, where S is the extrapolated signal per Neper (factor of e) of relaxation time. The nano-, micro-, and macroscale pore volume fractions multiplied by the skeletal porosity (P_A); Supplementary Table 2) produced the TD-NMR nano-, micro-, and macroscale porosities, respectively.

Supplementary Note 4: Small Angle X-ray Scattering (SAXS) analysis

SAXS data were collected with an area detector, corrected for background intensity, and radially averaged[9]. The resulting intensity $I(q)$, where q is the scattering length, was plotted for all measurements in Supplementary Figure 9, where the x-axis was chosen to represent q^2 and the y-axis to represent $q^2I(q)$. All curves were normalized by a constant factor, which led to an almost perfect

overlap of all curves at larger q. In this way, the scattering intensity is normalized to the same amount of coral material in all measurements. All data were very similar for $q > 1$ nm^{-1}, but showed some differences for smaller q-values. The intensity at larger q-data in Supplementary Figure 9 can be fitted by a straight line with the same slope for all specimens. This indicated that this scattering came from the bulk of the coral material and was unaffected by pH. The straight line in Supplementary Figure 9 is consistent with the existence of thin, plate-like materials, perhaps linked to the aragonite mineral. The fit in Supplementary Figure 9 (yellow line) corresponds to the expression reported inside the figure. In this expression, the value D represents the platelet thickness, according to Guinier's theory[10]. After subtraction from the measured scattering curves of the scattering from the thin platelets in the material, the remaining SAXS signal (which is attributed to porosity) was normalized to the same amount of coral skeletal material. The area under these SAXS curves was proportional to the pore volume per material volume. SAXS was only sensitive to small pores with a size roughly below 100 nm[9,10].

Supplementary References

1. Goffredo, S., Mattioli, G., Zaccanti, F. Growth and population dynamics model of the Mediterranean solitary coral *Balanophyllia europaea* (Scleractinia Dendrophylliidae). *Coral Reefs* **23**, 433-443 (2004).

2. Goffredo, S. *et al.* Biomineralization control related to population density under ocean acidification. *Nat. Clim. Chang.* **4**, 593-597 (2014).

3. Mehrbach, C., Culberson, C. H., Hawley, J. E., Pytkowicz, R. M. Measurement of the apparent dissociation constants of carbonic acid in seawater at atmospheric pressure. *Limnol. Oceanogr.* **18**, 897-907 (1973).

4. Dickson, A. G., Millero, F. J. A comparison of the equilibrium constants for the dissociation of carbonic acid in seawater media. *Deep-Sea Res. A* **34**, 1733-1743 (1987).

5. Dickson, A. G. Thermodynamics of the dissociation of boric acid in synthetic sea water from 273.15 to 298.15 K. *Deep-Sea Res. A* **37**, 755–766 (1990).

6. White, E.T., Tan, W.H., Ang, J.M., Tait, S., Litster, J.D. The density of a protein crystal, *Powder Technology* **179**, 55-58 (2007).

7. Pasquini, L. *et al*., Isotropic microscale mechanical properties of coral skeletons. *J. Roy. Soc. Interface* **12** (106) (2015).

8. Fantazzini, P. *et al.* Time-Domain NMR study of Mediterranean scleractinian corals reveals skeletal-porosity sensitivity to environmental changes. *Environ. Sci. Technol.* **47**, 12679-12686 (2013).

9. Pabisch, S., Wagermaier, W., Zander, T., Li, C., Fratzl, P. Imaging the Nanostructure of Bone and Dentin Through Small- and Wide-Angle X-Ray Scattering. *Meth. Enzymol.* **532**, 391-413 (2013).

10. Guinier, A., Fournet, G. *Small-angle scattering of X-rays* (Wiley, New York, 1955).
